# Contamination of *Fusarium proliferatum* and *Aspergillus flavus* in the Rice Chain Linked to Crop Seasons, Cultivation Regions, and Traditional Agricultural Practices in Mekong Delta, Vietnam

**DOI:** 10.3390/foods10092064

**Published:** 2021-09-01

**Authors:** Lien Thi Kim Phan, Trang Minh Tran, Kris Audenaert, Liesbeth Jacxsens, Mia Eeckhout

**Affiliations:** 1 Department of Food Technology, Safety and Health, Faculty of Bioscience Engineering, Ghent University, Coupure Links 653, 9000 Ghent, Belgium; MinhTrang.Tran@Ugent.be (T.M.T.); Liesbeth.Jacxsens@ugent.be (L.J.); Mia.Eeckhout@ugent.be (M.E.); 2 Faculty of Food Science Technology, Ho Chi Minh city University of Food Industry, 140 Le Trong Tan Street, Tay Thanh Ward, Tan Phu District, Ho Chi Minh City 700000, Vietnam; 3 Department of Plants and Crops, Faculty of Bioscience Engineering, Ghent University, Valentin Vaerwyck Weg 1, 9000 Gent, Belgium; Kris.Audenaert@ugent.be

**Keywords:** *Aspergillus flavus*, *Fusarium proliferatum*, rice chain, pre-harvest, post-harvest, Mekong Delta, seasons

## Abstract

This study evaluates the influence of crop seasons, cultivation regions, and traditional agricultural practices on the occurrence of *F. proliferatum* and *A. flavus* in the rice chain in the Mekong Delta, Vietnam. A survey on pre- and post-harvest practices was performed from 2017 to 2019 in parallel with sampling. Results showed that *F. proliferatum* (36.3%) and *A. flavus* (10%) were predominantly present throughout the rice chain. These fungi frequently occurred in winter–spring and autumn–winter crops in Can Tho paddy. Especially, *F. proliferatum* appeared both on the field and during transportation (50–100%), while *A. flavus* presented at all stages (10–33%). The occurrence of *F. proliferatum* reduced 70–27% after drying, depended on the seasons, compared to field and transportation stages and could not be detected anymore at further stages. Applying poor pre-harvest agricultural practices such as the use of certain varieties (Jasmine, DT8 varieties), combination of fertilizers (organic–inorganic), fields with crop debris, unhygienic boats, and delayed drying time of 8–12 h or 12–28 h resulted in an increase in fungal contamination on paddy. This study provides a detailed description of fungi contamination in crop seasons, cultivation regions, and agricultural practices, which may help in understanding the fungal dynamic and allow identification of good agricultural practices to mitigate the fungal contamination and potential mycotoxin production.

## 1. Introduction

Rice (*Oryza sativa* L.) is the foodstuff for 50% of the Asian population [1]. Rice is also the most important staple food in Vietnam. The average annual rice consumption of citizens is 218 kg per capita in 2017 [2], which is the main calorie intake in Vietnam. In Vietnam, rice is grown in the summer–autumn and autumn–winter crop seasons [3], in which frequent and heavy rainfalls occur, especially during harvest, making the rice crop prone to invasion by fungi [4].

*Aspergillus* is one of the dominant fungi in rice, especially *A. flavus* contaminating in rice of Malaysia [5], India [6], Philippines [7], and Vietnam [8]. *F. proliferatum* is also mentioned as the main contaminant, causing some diseases on rice crops, leading to a decrease in harvest yield and an increase in fumonisins accumulation in Japanese rice grains [9]. This fungus is able to survive on healthy rice grains under low temperatures for long periods [10]. *Fusarium* (21.8%) are also detected in paddy and rice in the Mekong Delta, Vietnam [8]. Pre- and post-harvest loss of the crop by the fungi is an emerging issue, especially in conducive tropical climates [6], such as Vietnam (e.g., high humidity, temperature, and drought). 

About 50% of Vietnamese rice production is produced in the Mekong Delta, in which Can Tho, Dong Thap, and An Giang provinces are the three main rice-growing provinces [11]. In this delta, a contract farming system was carried out on the basis of an agreement between the farmers and rice companies. In this contract, rice companies usually supply paddy variety, fertilizer, and knowledge and train farmers’ agricultural techniques and skills, while farmers must culture crops and follow rice companies’ instructions. After harvesting, paddy grown by farmers was transferred directly to the rice companies [12].

In addition to conducive environmental conditions, a relationship between agricultural practices and fungi contamination in rice with regard to the reduction of fungi in the rice chain has been reported by Goncalves et al. (2019) [10]. Vietnamese traditional pre-and post-harvest practices have been demonstrated to be associated with the increased contamination of maize with mycotoxins and *Fusarium verticillioides* in Vietnam [13,14] (e.g., crop residue management, selection of cereal variety, transport, etc.). However, as far as we know, no studies addressed the relationship between seasons, regions, and agricultural practices and contamination of *A. flavus* and *F. proliferatum* in the Mekong Delta rice chain in Vietnam. Thus, this study evaluates the contamination of *A. flavus* and *F. proliferatum* in the rice chain associated with (i) cropping seasons, (ii) cultivation regions, and (iii) traditional agricultural practices in the Mekong Delta, Vietnam, in order to be able to identify good agricultural practices to mitigate rice contamination. 

## 2. Materials and Methods

### 2.1. Study Location, Sampling, and Data Collection

**Figure 1:**
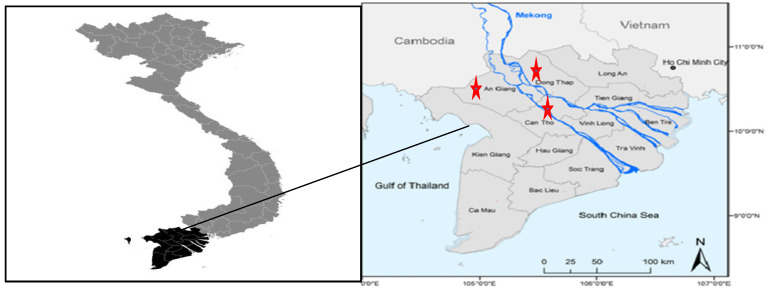
Sampling locations An Giang, Can Tho, and Dong Thap provinces of Mekong Delta (red stars), Vietnam.

The sampling plan was designed to get an insight into the variability amongst the sampling locations, seasons, and along the rice supply chain (Table 1). Two hundred and forty samples as paddy (*n* = 192) and white rice (*n* = 48) were collected in fields and companies from 2017 to 2019 in Can Tho, An Giang, and Dong Thap, which were considered as the nation’s top ten rice-producing provinces [11] (Figure 1).

Sampling was performed in five major rice crop seasons, including winter–spring from December 2017 to April 2018 (WS17-18), summer–autumn from April to August 2018 (SA18), autumn–winter season from August to November 2018 (AW18), winter–spring from December 2018 to April 2019 (WS18-19), and autumn–winter from August to November 2019 (AW19). Paddy and white rice samples from each farmer were collected at five different stages (*n* = 48 samples/stage) in the fields, after transporting from the fields to the factories, after drying for 30–60 min, after three months of storage, and white rice, produced after milling and cleaning the paddy (Table 1). Each sample was collected at nine points on the field and at drying stages, based on grid sampling, while for transport and storage stages, it was collected randomly from ten different containing bags, and for the milling stage, white rice was collected at different time points during the milling (i.e., 5, 10, 15, and 20 min).

The samples collected on the field stage were used to evaluate the impact of pre-harvest practices, such as choice of paddy variety (*n* = 48), fertilizer application (*n* = 48), and crop residue management (*n* = 48) on fungal contamination, while samples collected at transport stage can be considered to evaluate post-harvest practices as means of transportation (*n* = 48) and delayed drying time (*n* = 48) (Table 1). Each sample (2 kg) was stored in a polyethylene zipper bag, transported to Ho Chi Minh City University of Food Industry, Vietnam, and stored at 4 °C prior to further analysis.

### 2.2. Determination of Water Activity 

The water activity levels of 240 samples were measured using the EZ-200 (Freund, Tokyo, Japan). The instrument was calibrated at 25 °C with four calibration standards (e.g., MgCl_2_ (water activity: 0.328 a_w_), Mg(NO_3_)_2_ (0.529 a_w_), NaCl (0.753 a_w_), and NH_4_H_2_PO_4_ (0.930 a_w_)) supplied by the manufacturer. Each grain sample was measured three times after storage at 4 °C.

### 2.3. Isolation and Determination of Fungi

#### 2.3.1. Isolation and Identification by Macroscope and Microscope Observation

Five grains from each sample were directly plated on PDA (Potato Dextrose Agar, Merck, Germany) in 90 mm petri plates (Germany) and incubated at room temperature (25 °C) for 5–7 days. Each sample consisted of three replicates. The fungal colonies on grains were calculated and transferred to a new PDA plate. After 7-day incubation, fungi were examined macroscopically and microscopically (Optika, Italy). The different genera were then identified [15]. Afterwards, the fungi were sub-cultured on PDA and kept at 4 °C until molecular identification and aflatoxin production analysis of strains were conducted.

#### 2.3.2. Identification of Fungi by PCR Assay

Mycelium powder of each colony (400 mg) ground in liquid nitrogen was added to one mL of lysis buffer solution (CTAB 2.5% (Bio Basic Inc., Markham Ontario L3R 8T4 Canada), NaCl 5M (Merck, Darmstadt, Germany), tris HCl 1M (Himedia, Mumbai-, India), EDTA 1M, β-mercaptoethanol (Merck, Darmstadt, Germany)) and incubated at 65 °C for 1 h. Afterwards, 0.5 mL of solution (CTAB/Proteinase K (Meridian Life Science Inc., Memphis, TN, USA) 0.3 mg/mL 9/1: *v*/*v*) was added into mycelium powder–lysis buffer solution and incubated at 50 °C for 1 h. This solution was centrifuged (Hermle, 7326K, Wehingen, Germany) at 12,000 rpm/20 min. The supernatant (0.7 mL) was transferred to a new Eppendorf (2 mL) and was added 0.7 mL of solution (phenol (China)/chloroform (Merck, Darmstadt, Germany)/isoamyl-alcohol (Himedia, Mumbai, India) 25/24/1: *v*/*v*/*v*) and then centrifuged at 10,000 rpm for 5 min. The supernatant (0.7 mL) was transferred to a new Eppendorf, added 0.7 mL of solution (chloroform/isoamyl-alcohol 24/1: *v*/*v*), and centrifuged at 10,000 rpm/5 min. The supernatant (0.5 mL) was transferred to a new Eppendorf, added with 1 mL of ethanol (−20 °C, 99%), incubated at −20 °C overnight, and then centrifuged at 13,000 rpm/10 min. The supernatant was removed and washed with 0.5 mL of ethanol (70%), centrifuged at 13,000 rpm for 10 min, and re-washed twice. After removing the supernatant, the residue was dried at room temperature (25 °C) and then was dissolved with 0.15 mL of TE 1X solution and stored at −20 °C.

DNA amplification carried out in an Agilent Technological PCR (Santa Clara, CA, USA). Fifty µL of PCR-reaction mixture consisted of 0.25 µL Dream Taq polymerase (500U, Thermo Scientific, Waltman, MA, USA), 5 µL Dream one Taq buffer 10X including 20 mM MgCl_2_ (Thermo Scientific, Waltman, MA, USA), 0.1 µL each dNTP (10 µm, VWR International, Leuven, Belgium), 1 µL forward primer, ITS4 (400 mM, IDT, Queenstown, Singapore) (5′-TCCTCCGCTTATTGATATGC-3′), 1 µL reverse primer ITS5 (400 mM, IDT, Queenstown, Singapore) (5′-GGAAGTAAAAGTCGTAACAAGG-3′), 38.35 µL of DNA and ultra-clean water (DEPC Water, ABT, Ha Noi, Vietnam), and 4 µL of DNA sample (diluted 10x in DEPC Water). The PCR conditions set up followed: initial denaturation at 95 °C for 6 min, followed by 40 cycles of 30 s at 95 °C, 45 s at 55 °C, 45 s at 72 °C, and a final extension at 72 °C/5 min. The PCR products were dyed with a 6X G, gel red (ABT, Ha Noi, Vietnam), separated on an agarose gel 1.8% (*w*/*v*) at 100 V/45 min, and visualized on a Bio-Print TX4 VILBER LOURMAT. Following PCR, amplicons were cleaned using the E.Z.N.A.^®^ Cycle-Pure Kit (VWR International, Leuven, Belgium). The purified PCR products were sent to 1st BASE DNA (Apical Scientific SDN BHD, Selangor, Malaysia) for sequencing by Sanger sequencing. The identification of fungal names was determined by doing BLAST on NCBI (National Center for Biotechnology Information) database (https://blast.ncbi.nlm.nih.gov/BLAST, accessed from June to December 2020).

### 2.4. Determination of Mycotoxigenic Fungi by LC–MS/MS

#### 2.4.1. Extraction of Mycotoxins from Fungi

*A. flavus* and *F. proliferatum* isolates were cultured on rice medium (2% agar-VWR, BDH chemicals; 2% rice powder) at 30 °C/10 days. Five grams of rice medium (using cork borer 4 mm to collect medium) containing fungi were added to previously weighed 15 mL tubes. Aflatoxin B1 and Fumonisin B1 were extracted with 2.5 mL of chloroform (HPLC grade, VWR International, Leuven, Belgium), vortexed (VM-1000) for 1 min, shaken 200 rpm/60 min (S100H, Elmasonic, AHRD), and then centrifuged at 3000 rmp/5 min (EBA200). The supernatant was transferred to a 5 mL glass tube and dried gently under 40 °C by nitrogen (MRC, AHRD). One mL mobile phase MeOH: H_2_O (0.1% acid formic) 7/3: *v*/*v* was added to such dried glass tube and vortexed. This solution was filtered by syringe filters 0.45 μm (PTFE syringe filter, Thermo Fisher) and transferred to a new vial. 

#### 2.4.2. LC–MS/MS

Detection of aflatoxin B1 and fumonisin B1 from *A. flavus and F. proliferatum*, cultured on rice medium at 30 °C/10 days, respectively, was carried out using LC–MS/MS (SHIMADZU 8040, Tokyo, Japan) in positive electrospray ionization (ESI+) mode. Mycotoxins detected were analyzed in 70% mobile phase A (methanol (Merck, Darmstadt, Germany) and 30% mobile phase B (water with 0.1% formic acid (*v*/*v*) (Merck, Darmstadt, Germany). The sample chamber and column were maintained at 15 °C, and 10 µL of the sample was injected. The total analysis time was 8 min/sample. The column was washed with methanol (500 µL/sample). Chromatographic separation was performed in Shim-pack XR-0DSII (75 m × 2 mm, 2.2 μm) column with a guard column (4.6 × 12.5 mm and 5 µm, Agilent, Santa Clar, CA, USA), and flow rates were 0.5 mL/min. The capillary voltage was at 1.96 kV, and source and desolvation temperatures were 240 °C and 350 °C, respectively.

### 2.5. Pre- and Post-Harvest Practices in the Vietnamese Mekong Delta Rice Chain

A survey was conducted by means of a face-to-face interview with a structured questionnaire to understand Mekong Delta pre- and post-harvest traditional agricultural practices (Table 2). Forty-eight participants, including farmers and rice processing companies, donating paddy or rice samples in Can Tho, Dong Thap, and An Giang province of the Mekong Delta, were interviewed when collecting samples. The interview focused on the pre-harvest activities as a choice of variety, the approach to prepare soils, the fertilizer used, and the approach to managing crop residues. Post-harvest practices included means to transport paddy from field to factory, delayed drying time (calculated from harvesting to drying), and drying conditions (temperature and time drying), which were reported to be related to the occurrence of fungi on agricultural products [13,14,16]. Additionally, to obtain additional objective evidence, registration forms and documents related to rice production were checked by the investigators during the interview.

### 2.6. Data Analysis

All the descriptive and statistical analyses were done using Chi-square test with significance level of α = 0.05 to assess the impact of the five crop seasons, three cultivation regions, and the pre- and post-harvest practices on the occurrence of *A. flavus* and *F. proliferatum*. The water activity of 240 samples collected in the Mekong Delta at five stages (48 samples/stage) was analyzed using one-way ANOVA in IBM SPSS Statistic version 20.0, (IBM Corp., Armonk, NY, USA). Data of the interview in this study were analyzed by Microsoft Excel 2013 (Redmond, WA, USA). 

## 3. Results and Discussion

### 3.1. Agricultural Practices in the Vietnamese Mekong Delta 

The results of the face-to-face interview with farmers and rice processing companies on the currently applied traditional agricultural practices in rice production in the Mekong Delta are shown in Table 2. With regard to paddy variety, the selection criteria of rice varieties of the Mekong Delta farmers are high yield, high resistance to disease, and high market price, resulting in the rice lines IR50404, OM5451, DT8, and Jasmine, selected to grow in the delta. Ninety percent of farmers applied inorganic fertilizer during paddy production to improve soils and crops, whereas few growers (10%) utilized a combined fertilizer (inorganic and organic) due to inefficiency in crop yield and expensive price. With respect to crop residue management, farmers managed the crop debris by removing them (42%) to use them for mushroom cultivation or animal feed, by burning (44%) to destroy the crop residues to ashes to supply nutrients for the soil, and by bio-decomposer, containing a set of beneficial microorganisms, producing enzymes such as cellulose, amylase, hemicellulase, lignin, and protease, etc., which decompose organic substances into nutrients in order to improve field soil (e.g., AT Bio-decomposer^®^). In the Mekong Delta, because the network of rivers and canals is interlaced, trucks and boats are two popular vehicles used to transport paddy from the fields to the factories. Of those, 52% of the farmers used trucks, while 33% used boats and 15% combined both. However, both means were not cleaned before loading the paddy. For delayed drying time, because transportation took a long time, especially in the rainy seasons, and dry machines did not afford to dry all paddy in harvest seasons, the drying time of paddy was delayed. In this delta, paddy was dried by drying machines to reduce moisture content at 40–45 °C/18–20 h, and rice companies tested the moisture content of the paddy grain by using a variety of measures (e.g., biting and moisture content-measuring machines) after different delayed drying times: 2–8 h, 8–12 h, and 12–28 h.

### 3.2. Water Activity 

The water activities of 240 samples collected from three regions (Can Tho, Dong Thap, and An Giang) between December 2017 and December 2019 at five stages (48 samples/stage) are shown in Table 3. The data indicated a significant difference in the water activity (a_w_) of grains before and after drying (*p* < 0.05). The higher mean a_w_ were found in samples of the field stage (a_w_ 0.95 ± 0.03) and of transporting stages (a_w_ 0.95 ± 0.03) when comparing to samples of drying stage (a_w_ 0.72 ± 0.09), storage stage (a_w_ 0.71± 0.07), and milling stage (a_w_ 0.69 ± 0.05). However, the water activity of grains amongst seasons and cultivation regions at the same stage didn’t show a significant difference (*p* > 0.05) (data are not shown in this study).

### 3.3. Fungi Contamination in the Vietnamese Mekong Delta Rice Chain

**Figure 2:**
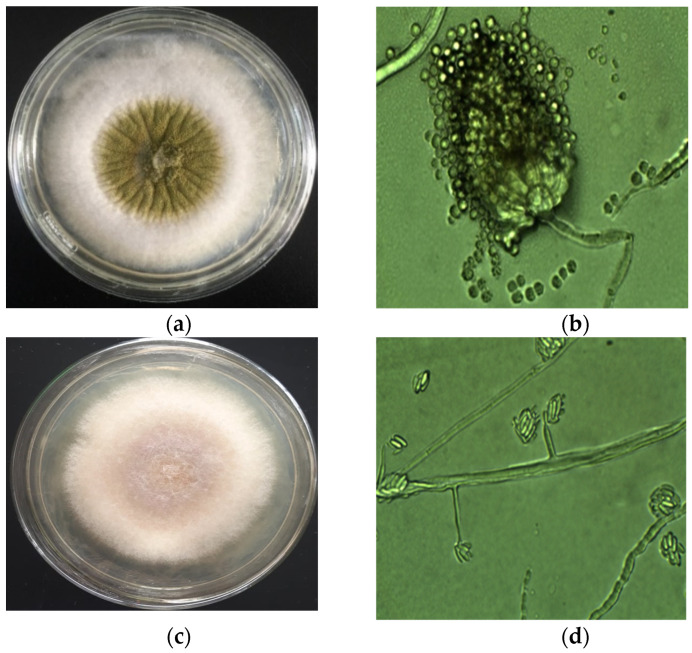
*A. flavus* on PDA (**a**), microscope (**b**), and *F. proliferatum* on PDA medium (**c**) and microscope (**d**).

The fungi from the paddy (*n* = 192) and white rice (*n* = 48) samples collected in the Mekong Delta from December 2017 to December 2019 were isolated and grouped by morphological characteristics by macroscopic and microscopic observation (e.g., *A. flavus* and *F. proliferatum* in Figure 2); such fungi were confirmed by molecular analysis with primers of ITS 4 and 5.

**Figure 3:**
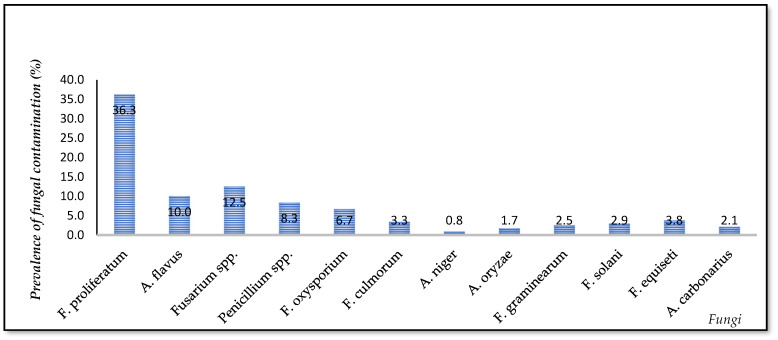
Prevalence of samples contaminated by fungi in the Vietnamese rice chain (based on characterization of isolates).

Figure 3 indicates that the dominant genera in the Mekong Delta rice chain were *Fusarium* spp., *Aspergillus* spp., and *Penicillium* spp. Of those, 12 types of fungal species were detected as *F. proliferatum, A. flavus, Fusarium* spp., *Penicillium* spp., *F. oxysporum*, *F. equiseti*, *F. culmorum*, *F. solani*, *F. graminearum*, *A. carbonarius*, *A. oryzae*, and *A. niger. F. proliferatum* was found most frequently (36.3%), followed by *A. flavus* (10%) and other fungi (0.8–12.5%). In total, 1334 isolates were identified, such as *F. proliferatum* (889 isolates), *A. flavus* (50 isolates), and other fungi (395 isolates). The relative density of *F. proliferatum* was higher than *A. flavus* and other fungi regardless of factors. AFB1-positive *A. flavus* and FB1-producing *F. proliferatum* grown on rice medium at 30 °C/10 days were particularly high, 44% (*n* = 15/34) and 60% (*n* = 529/889), respectively; isolated from paddy but from white rice, only AFB1-positive *A. flavus* was detected, 31% (*n* = 5/16). The AFB1 and FB1 levels extracted from the fungi cultured on rice medium were from 1.13 to 508 µg/kg and from 13 to 3455 µg/kg, respectively.

#### 3.3.1. Contamination of *A. flavus* and *F. proliferatum* by Crop Seasons

To evaluate the occurrence of *A. flavus and F. proliferatum* by crop seasons, samples of paddy and white rice from five crop seasons WS17-18 (*n* = 75), SA18 (*n* = 35), AW18 (*n* = 10), WS18-19 (*n* = 50), and AW19 (*n* = 70), collected from three provinces of the Mekong Delta, were analyzed.

**Figure 4:**
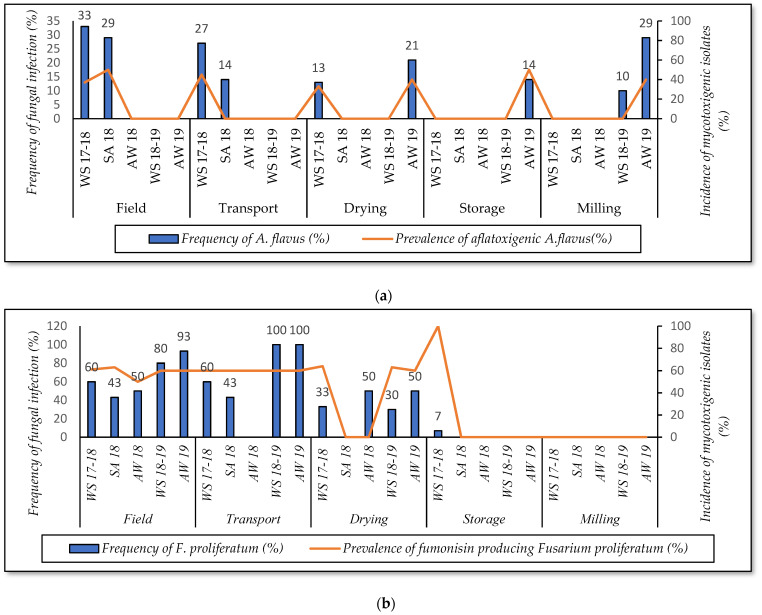
Percentage of *A. flavus* (**a**) and *F. proliferatum* (**b**) contaminated rice samples and mycotoxigenic isolates in five seasons over the rice supply chain in Vietnam. WS17-18—winter–spring from December 2017 to April 2018; SA18—summer–autumn from August to November 2018; AW18—autumn–winter season 2018; WS18-19—winter–spring from December 2018 to April 2019; AW19—autumn–winter from August to November 2019.

At the field and transportation stage, Figure 4a shows that *A. flavus* was detected at a high frequency on paddy collected in the WS17-18 and SA18, whereas this fungus was absent in the other seasons. The strain was frequently isolated from the WS17-18 (27–33%), with the incidence of aflatoxigenic isolates ranging from 37 to 45%. These rates were lower in the SA18 (14–29%) (Figure 4a). *F. proliferatum* was the predominant species on paddy of the AW19 (93–100%), followed by WS18-19 (80–100%) and WS17-18 (60%). In these seasons, the prevalence of isolates producing fumonisin B1 was also high, ranging from 60 to 61% (Figure 4b). In this study, seasons significantly impact the contamination of *A. flavus* and *F. proliferatum* on paddy at these stages (*p*-value < 0.05).

At the drying stage, the data revealed a higher prevalence of *A. flavus* contamination on paddy grains in the AW19 (21%) when compared to the paddy of the WS17-18 (13%). This species was not detected in other seasons. Similarly, a higher percentage of AFB1-producing *A. flavus* was found on the paddy of the AW19 rather than on the paddy of the WS17-18 (40% vs. 33%). *F. proliferatum* infection on paddy ranged from 30 to 50% in most seasons (except for SA18). Interestingly, the incidence of this strain reduced dramatically between 27 and 70% depending on the seasons, compared to previous stages. However, the impact of crop seasons on the occurrence of both strains on paddy in this stage was not clearly different (*p*-value > 0.05).

At the storage and milling stage, Figure 4a shows that the percentage of the samples infected by *A. flavus* was 14% in the AW19 at the storage stage. At the milling stage, the incidence of such fungal contamination was 29% and 10% on white rice in the AW19 and WS18-19, respectively, whereas paddy from the other seasons was not contaminated. Remarkably, the prevalence of *F. proliferatum* contamination was not detected in all seasons (except for WS17-18 at the storage stage) (Figure 4b) (*p*-value > 0.05).

Overall, *A. flavus* was frequently found in the winter–spring and autumn–winter crop seasons throughout the rice chain, while *F. proliferatum* occurred high in these seasons from field to transportation, reduced significantly at the drying, and disappeared in further stages. 

#### 3.3.2. Infection of *A. flavus* and *F. proliferatum* by Cultivation Regions

To assess the effect of cultivation areas on the contamination of *A. flavus and F. proliferatum*, paddy and white rice in Dong Thap, Can Tho, and An Giang province were collected and analyzed.

**Figure 5:**
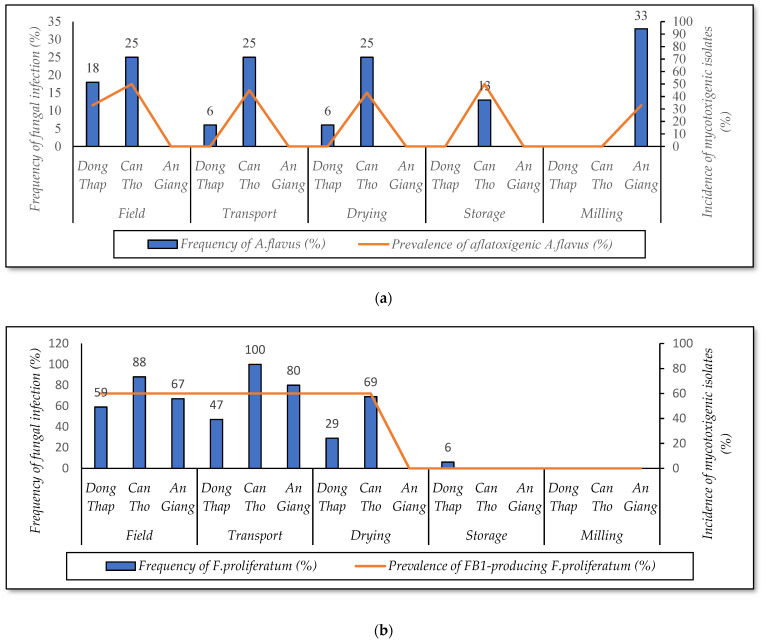
Percentage of *A. flavus* (**a**) and *F. proliferatum* (**b**) contaminated samples and mycotoxigenic isolates in different locations in Mekong Delta.

Figure 5b shows that at the fields and transportation stage, paddy in Can Tho was contaminated most frequently by *F. proliferatum* (88–100%), followed by An Giang (0–67%), and Dong Thap (47–59%). For *A. flavus*, the highest frequency of the infected grains was also found in the Can Tho (25%), followed by Dong Thap (6–18%), and this fungus disappeared in An Giang paddy. Additionally, AFB1-producing *A. flavus* was found highest in Can Tho (Figure 5a). With regard to the density of fungi, *F. proliferatum* was the dominant species on paddy of these stages across regions (57–80%), followed by other fungi (20–40%), and then *A. flavus* (0–6%) (data were not shown in this study). Moreover, cultivation regions strongly impacted the occurrence of such strains (*p*-value < 0.05).

After drying, the frequency of Can Tho (*n*= 16) and Dong Thap (*n* = 17) paddy containing *A. flavus* was 25% and 6%, respectively, while this strain was absent in An Giang grains (*p*-value > 0.05). The higher rates were detected in *F. proliferatum* contamination for Can Tho (69%) and Dong Thap (29%) (*p*-value > 0.05). Importantly, contamination of *F. proliferatum* reduced significantly, more than 30–40%, compared to that at the field and transportation stage, but the percentage of mycotoxin producing isolates was still high (60%) (Figure 5b).

Figure 5a shows that after three months of storage in the warehouse, the percentage of *A. flavus* contamination on Can Tho paddy was 13%, with a high incidence of AFB1-positive isolates of 50%. The data revealed that at the milling stage, the appearance of *A. flavus* was detected only on An Giang paddy (33%) (*p*-value > 0.05) with AFB1-producing isolates (33%). In contrast, in general, infection of *F. proliferatum* was absent at the storage and milling stage. 

The highest contamination with *A. flavus* and *F. proliferatum* was observed on the paddy of the Can Tho region. *A. flavus* was found throughout the rice chain, whereas *F. proliferatum* was detected more frequently at the field and transportation stages and was really low and/or absent at drying, storing, and milling stages in the winter–spring and autumn–winter crop seasons.

### 3.4. Impact of Pre-Harvest Practices on the Infection of F. proliferatum and A. flavus 

#### 3.4.1. Selection of Paddy Variety

**Figure 6:**
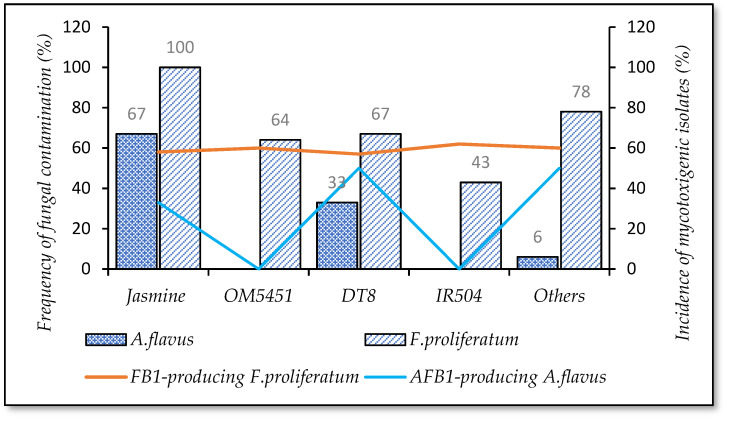
Incidence of fungal contamination and mycotoxigenic fungi in different paddy lines.

Figure 6 indicates that the highest incidence of *A. flavus* was detected on the line Jasmine (67%), followed by the DT8 (33%) and other lines (6%), and this fungus was absent in the two lines OM5451 and IR504. A higher incidence of AFB1-positive isolates was observed in the DT8—other paddy varieties (50%), compared to Jasmine (33%) (Figure 6). Similarly, the Jasmine paddy line showed the highest frequency of *F. proliferatum* contamination (100%). This rate was slightly lower in other paddy varieties, DT8, OM5451, and IR504 (Figure 6). The percentage of FB1-producing isolates was found high on all paddy varieties, ranging from 57 to 62%. Overall, the fungal infection among paddy varieties was not clearly different for both fungi (*p*-value > 0.05).

#### 3.4.2. Crop Residue Management

**Figure 7:**
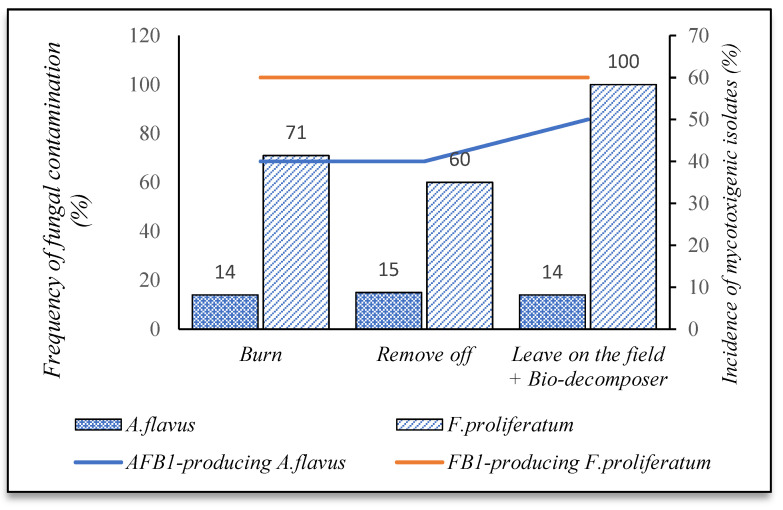
Incidence of fungal contamination and mycotoxigenic fungi in function of crop residue management practices.

Figure 7 indicates that fields with crop residues, which were decomposed by bio-decomposer (e.g., AT Bio-decomposer^®^) sprayed by farmers after harvesting crops, were more susceptible to infection of *F. proliferatum*. Indeed, the incidence of this fungus was detected in the greater number of such fields (100%, *n* = 7), while paddy of the fields, free crop residues by burning (71%, *n* = 21) and removing off (60%, *n* = 21) was contaminated less. The data indicated that the occurrence of *F. proliferatum* in the fields, free crop residues reduced from 30 to 40%, compared to the field containing crop residues. However, the frequencies of FB1-producing isolates on paddy were high across all three approaches (60%) (Figure 7). By contrast, contamination of *A. flavus* on paddy was not clearly different among crop residue managements ranging from 14% to 15%, with an incidence of aflatoxigenic isolates of 40–50% (Figure 7). Crop residue management did not significantly impact the occurrence of both fungi on paddy (*p*-value > 0.05).

#### 3.4.3. Fertilizer Application 

**Figure 8:**
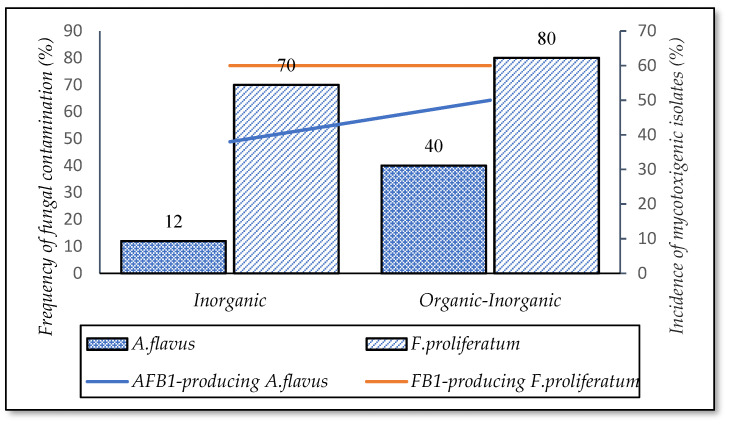
Incidence of fungal contamination and mycotoxigenic fungi in fertilizer applications.

A majority of the fields using a combined fertilizer (inorganic and organic) (80%, *n* = 5) were contaminated by *F. proliferatum* (Figure 8). This percentage was lower for the fields using inorganic mono-fertilizer (70%, *n* = 43), but the percentage of FB1-producing isolates was not different for both methods (60%) (Figure 8). Likewise, the fields applying combined fertilizer had the highest contamination with respect to *A. flavus* (40%). Such prevalence was lower for the fields using inorganic only (12%). Of those, *F. proliferatum* was the predominant species on paddy of both methods (86–98%) (data were not shown in this study). The occurrence of both fungi was independent of the fertilizer applications (*p*-value > 0.05).

### 3.5. Impact of Post-Harvest Practices on the Contamination of F. proliferatum and A. flavus 

#### 3.5.1. Means of Transportation

**Figure 9:**
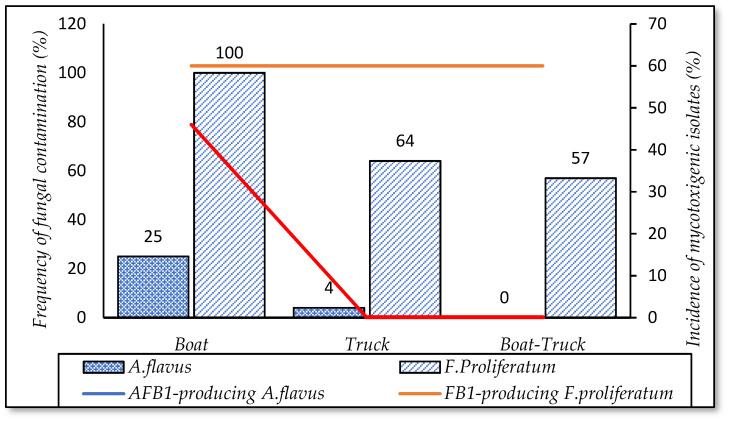
Incidence of fungal contamination and mycotoxigenic fungi in means of transportation.

The data showed that 100% of paddy transported with boats (*n* = 16) was infected by *F. proliferatum*. This rate was lower for truck transport (64%, *n* = 25) and a combination of both vehicles (57%, *n* = 7) (Figure 9). Figure 9 demonstrates that the incidence of isolates producing FB1 does not depend on means of transport (60%). Similar to *F. proliferatum* contamination, a higher frequency of *A. flavus* infection was found on paddy, using boats (25%), compared to trucks (4%), and this fungus was not detected on paddy transported with a combination of both. The paddy contamination of both strains was influenced by means of transportation (*p*-value < 0.05).

#### 3.5.2. Delayed Drying Time

**Figure 10:**
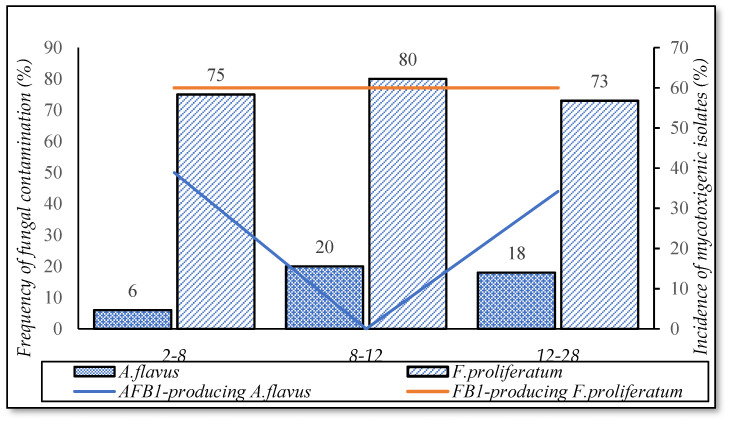
Incidence of fungal contamination and mycotoxigenic fungi in function of delayed drying times.

Figure 10 pinpoints that the occurrence of *F. proliferatum* found in paddy with delayed drying of 8–12 h (80%), and of 12–28 h or 2–8 h, was ranging between 73–75%, and the incidence of FB1- positive isolates was 60%. Paddy dried after 12–28 h and 8–12 h was contaminated higher by *A. flavus* (18% to 20%) than 2–8 h (6%) (*p*-value > 0.05). However, the percentage of AFB1-producing *A. flavus* was observed at 12–28 h and 2–8 h.

## 4. Discussion

The presence of fungi in the rice chain not only results in grain losses but also pose a threat to consumer health, as various fungi are capable of producing mycotoxins [17]. To date, no study of fungal contamination in the Vietnam Mekong Delta rice chain has been reported yet. Notwithstanding, there are some prior reports on mycotoxin and fungal contamination in stored or marketed rice [18,19,20] 

The data revealed that paddy and white rice were contaminated by fungal species, *F. proliferatum, A. flavus, Aspergillus* spp., *Fusarium* spp., etc. *F. proliferatum* was a predominant species in the rice chain (Figure 3). Trung et al. (2001) [8] reported that paddy harvested in the Mekong Delta was highly contaminated with *Fusarium* (21.8%). Other studies also mentioned that *Fusarium* spp. were found most frequently on rice. Samples were contaminated by *Aspergillus flavus* (10%), which is in accordance with other investigations [10,21,22,23,24]. For instance, twenty-five rice samples in the Mekong Delta were infected by *Aspergillus* with the highest incidence (43.75%) [8]. Additionally, *Aspergillus* appeared commonly in 36 stored rice samples collected in India [25].

*A. flavus* and *F. proliferatum* strains are well-known as aflatoxin B1 (AFB1) and fumonisin B1 (FB1) producers in agricultural products [4,26]. In this study, *A. flavus* (50 isolates) and *F. proliferatum* (889 isolates) identified in the rice chain were used to assess their ability to produce AFB1 and FB1 on rice medium. Of those, 44% and 31% of AFB1-positive isolates were isolated from paddy and white rice, respectively, and 60% of isolates producing FB1 were isolated from paddy. The AFB1 and FB1 levels produced by the fungi on rice medium ranged from 1.13 to 508 µg/kg and 13 to 3455 µg/kg, respectively. Our results are lower or higher than those reported by Sales and Yoshizawa (2005) [7] in paddy and polished rice in the Philippines, who reported that 78% and 28% of *A. flavus* on paddy and polished rice, respectively, had the ability to produce aflatoxins with a large range of concentration (<0.025–371 µg/kg and 2–2589 µg/kg, respectively). This difference could be related to fungal strains, medium, and incubation conditions.

Regarding *A. flavus* and *F. proliferatum* contamination associated with crop seasons, Figure 4 indicates that these fungi were usually identified in the winter–spring and autumn–winter seasons. According to previous reports, rice in tropical Asia was predominantly contaminated by *A. flavus* [6], which grow at 25–30 °C on paddy [27] and 25–35 °C on white rice and brown rice [28], and by *F. proliferatum* [29] developing at 25–30 °C [30] and 20–30 °C [31]. In the Mekong Delta, the winter–spring crop lasts from December to April next year, in which the temperature is about 23–35 °C. The autumn–winter crop lasts from August to November with temperatures ranging 22–30 °C [3]. These conditions led to fungal growth quickly. Another explanation is that, in winter–spring, most fields are in the invasion of the seawater and drought conditions [32]. Some paddy lines were not salt- and drought-tolerating paddy varieties, resulting in vulnerability to crop disease (e.g., *Fusarium* diseases) [33], resulting in an increase in fungi infection. In contrast, in the summer–autumn crop or autumn–winter crop, fields are not in the invasion of the seawater and drought conditions; as a result, crops were not susceptible to *Fusarium* diseases. 

In terms of the fungal infection related to rice cultivation areas, the data indicated that fungal contamination throughout the rice chain had significant differences. This is because current traditional agricultural practices result in appropriate conditions for fungal proliferation [29]. In fact, paddy from Can Tho was highly contaminated by fungi since farmers in this province grew only one paddy variety (e.g., Jasmine or DT8) for all seasons such as winter–spring, summer–autumn, and autumn–winter per year. In winter–spring, some Can Tho fields are in the invasion of the seawater and drought conditions [32]. The varieties such as Jasmine or DT8 do not have the ability to tolerate seawater and drought well, bringing about vulnerability to crop disease. This is in agreement with other works, which suggested that drought conditions were associated with an increase in Fusarium diseases and fungal growth [33]. The growers in the An Giang and Dong Thap region cultured other varieties (e.g., OM5451, IR50404, etc.), which are more resistant to drought problems than Jasmine and DT8 varieties based on the survey. The fields of these regions are not invaded by seawater, leading to crops with a good ability to resist diseases resulting in a low fungal appearance on the paddy. Therefore, to mitigate contamination of fungi on the rice chain, Mekong Delta farmers should avoid planting mono-paddy variety for all seasons, especially avoiding using paddy lines, which are vulnerable to extreme environments such as drought.

The data in this current work indicated that pre- and post-harvest practices were the key drivers for fungi contamination on paddy. Although the availability of good agricultural practice guidelines by FAO [34], the Mekong Delta farmers have not yet recognized the role of the agricultural practices in the mitigation of fungal infection in the rice chain. The data showed that the occurrence of fungi throughout the rice chain was the consequence of poor pre- and post-harvest activities such as selection of paddy variety, fields with crop debris, mixed fertilizer use, unhygienic boats, and delayed drying time. It means that the rice-producing industry should use these findings as good agricultural practices to reduce fungal and potential mycotoxin contamination, resulting in a better food safety governance from farm to fork. It also helps farmers to acknowledge and apply good agricultural practices if their customers (the rice industry) require them.

Regarding paddy varieties, to make the crop less vulnerable to fungal contamination, it is essential to limit all factors impacting the growth of plants, such as soils, climates, and agricultural practices, etc. The results showed that Jasmine and DT8 varieties were more prone to *Aspergillus flavus* and *F. proliferatum* than other paddy lines. This could be related to their ability to tolerate salt and drought issues. These paddy lines did not tolerate seawater and drought well, as mentioned above. Some growers cultured Jasmine and DT8 in late December in the winter–spring crop. As a result, these paddy lines were exposed to saltwater intrusion and drought issues at the end of the crops [32], leading to an increase in crop diseases [33]. Therefore, farmers and rice companies should select appropriate paddy lines for crop seasons and locations to reduce fungal contamination.

Discussing crop residue management, in the present work, 14% of Mekong Delta farmers left crop residues on the fields and used bio-decomposer (e.g., AT Bio-decomposer^®^), containing a set of beneficial microorganisms producing enzymes such as cellulose, amylase, hemicellulase, lignin, protease, etc., which quickly decompose organic substances (e.g., crop debris) into nutrients to improve field soil. Figure 7 shows that the fields without crop residues by burning or removing off had a lower incidence of *F. proliferatum* than fields containing crop debris. This is not surprising because crop debris on the field was decomposed by microorganisms to organic matter, containing high in organic carbon and nitrogen concentrations, stimulating fungal pathogens in the soils to proliferate [35]. Moreover, crop residues on the fields are supposed to be the main sources of inoculum fungi for the next crop seasons [13,36,37]. Thus, management of crop residues by burning and removing them could be considered an effective method for mitigation of *F. proliferatum* contamination on paddy. Although burning after harvest decreased fungi contamination, this negatively impacts the environment. Meanwhile, removing off crop debris can be a reasonable alternative since it minimizes *F. proliferatum*, and straws after removing are usually used for various objectives such as animal feed, biofuels, mushroom cultivation, etc.

For fertilizer application, fertilizer stimulates the growth of the microbial community in the soils and the crop yields. The data showed that *Aspergillus flavus* and *F. proliferatum* contamination were higher in the mixed fertilizer fields than inorganic fields. This could be explained that the mixed fertilizer (organic/inorganic: 30/70 *v*/*v*) containing high nitrogen contents led to an increase in the microbial community, especially the fungal population in the soils [38]. Organic matter could have supported fungal activity and fungal growth [35]. In the meanwhile, the fields added by inorganic had lower organic carbon contents, making a lower incidence of fungi [38].

Based on the survey, 33% of rice companies in the Mekong Delta used boats to transport paddy from fields to factories. The data revealed that a higher incidence of *F. proliferatum* and *A. flavus* was observed on paddy transported by boats, compared to that from trucks or/and combinations. Because boats were used to transport paddy from the fields, which were far from factories or companies, it took a lot of time. As a result, such paddy was exposed to the airborne transmission of *F. proliferatum* and *A. flavus*. Moreover, boats were wet and unhygienic vehicles containing paddy from other fields, dirt, crop residues, etc., which were potential fungal sources [39]. To prevent contamination of fungi, rice companies or farmers should use hygienic means of transport. 

According to information collected from the survey, after harvest, the paddy was transported by different means (e.g., trucks and boats). Transportation took a long time due to the rainy seasons. Moreover, dry machines did not afford to dry all paddy in harvest seasons, so the drying time of paddy was delayed. Figure 10 shows that a higher percentage of *F. proliferatum* and *Aspergillus flavus* contamination was observed at 8–12 and 12–18 h. It is because such paddy had a high water activity (0.95 ± 0.03 a_w_—Table 3), which was the optimal condition for fungi to grow [40], since *A. flavus* can grow at a water activity of 0.82–0.99 [41], and most strains of *Fusarium* spp. develop at water activity from a_w_ 0.854–0.99 [30]. It could prolong the exposure period of the paddy grains to environments (e.g., airborne pathogens), domesticated animals, insects, rodent animals (soilborne fungal pathogens), etc., when delaying drying time. This result is consistent with studies and recommendations on safe drying conditions of grains to minimize fungi and mycotoxin. Drying should be carried out quickly after harvest to inhibit fungal growth and mycotoxin production [42,43]. Hence, minimizing the periods between harvesting and drying should be applied in post-harvest management to decrease fungal contamination.

Contamination of fungi on paddy was dramatically different from rice chain stages regardless of seasons and localities. For instance, the prevalence of fungi-positive samples was high in the field and transportation stage and low in the further stages with respect to *F. proliferatum*. Because in the field and transportation stage, the water activity in the paddy was quite high (a_w_ 0.95 ± 0.03) (Table 3), being favorable for fungi to proliferate. This is similar to the study in Nigeria; field paddy was contaminated by fungi higher than that in preservation [44]. A higher incidence of *F. proliferatum* was associated with fungal diseases on the field [45]. *A. flavus*, however, was detected throughout the rice chain due to ideal tropical weather, improper storage [46], inappropriate facilities, and poor technical conditions [47,48]. At the milling stage (white rice), the fungi were detected lower than at former stages since they were distributed mainly in the husk, brown rice, and bran [49], but during milling, these components (containing nutrients for fungi to grow and produce mycotoxins) were removed, and contamination of fungi on white rice was low. Hence, the most significant step to overcome fungal pathogen is the removal of bran to obtain white rice during the milling process [49].

## 5. Conclusions

*F. proliferatum* and *A. flavus* were frequently found in the rice chain in the Mekong delta. Crop seasons, cultivation regions, and agricultural practices (pre-harvest: paddy variety, crop residue management, and fertilizer application; post-harvest: means of transportation and delayed drying time) were the main factors for the increase in fungal contamination in the rice chain. These findings could be used as a reference for the mitigation of mycotoxigenic fungi in pre- and post-harvest in the rice chain in the Mekong Delta, Vietnam. It means that the rice-producing industry and farmers could use the results as good agricultural practices to reduce fungal and potential mycotoxin contamination, leading to food safety from the farm to the plate. It also helps farmers recognize and apply such good agricultural practices if their customers (the rice industry) require them as a prerequisite.

## Figures and Tables

**Table 1:** Sampling plan (*n* = 240), distributed over the crop seasons (WS—winter–spring; SA—summer–autumn; AW—autumn–winter) and three growing regions (Dong Thap, Can Tho, and An Giang) along the Mekong Delta rice chain between 2017 and 2019.

Stages in the Rice Chain	Dong Thap (*n* = 85)					Can Tho (*n* = 80)			An Giang (*n* = 75)				Total Samples/Stage
	WS17/18	SA18	AW18	WS18/19	AW19	WS17/18	WS18/19	AW19	WS17/18	SA18	WS18/19	AW19	
Field	7	3	2	3	2	5	4	7	3	4	3	5	48
Transport	7	3	2	3	2	5	4	7	3	4	3	5	48
Drying	7	3	2	3	2	5	4	7	3	4	3	5	48
Storage	7	3	2	3	2	5	4	7	3	4	3	5	48
Milling	7	3	2	3	2	5	4	7	3	4	3	5	48
Total	35	15	10	15	10	25	20	35	15	20	15	25	240

**Table 2:** Main pre-and post-harvest practices in paddy production in Mekong Delta, Vietnam.

Agricultural Practices	Applications	2017–2019
		Total (*n* = 48) %
Pre-harvest practices		
Paddy varieties	OM 5451	23
	IR 50404	14
	DT 8	13
	Jasmine	13
	Others (OM4900, 7347, ST24, Timthan)	37
Fertilizers	Inorganic	90
	Organic–Inorganic (30/70:*w*/*w*)	10
Crop residue management	Burn	44
	Remove off	42
	Leave on the field and use bio-decomposer to decompose crop debris	14
Post-harvest practices		
Means of transportation	Trucks	52
	Boats	33
	Boats–Trucks	15
Delayed drying duration (hours)	2–8	67
	8–12	10
	12–28	23

**Table 3:** Water activity of paddy and white rice (*n* = 240) (Mean ± SD) in Mekong Delta in 2017–2019.

Describe Statistics	Field (*n* = 48)	Transport (*n* = 48)	Drying (*n* = 48)	Storage (*n* = 48)	Milling (*n* = 48)
Mean ± SD	0.95 ± 0.03 ^a^	0.95 ± 0.03 ^a^	0.72 ± 0.09 ^b^	0.71 ± 0.07 ^b^	0.69 ± 0.05 ^b^
Median	0.96	0.96	0.71	0.68	0.68
Min	0.87	0.88	0.53	0.56	0.63
Max	0.98	0.98	0.92	0.91	0.80

Water activity was measured at 25 °C; different letters ^a,b^ point out a significant difference using one-way ANOVA at a significant level of α = 0.05.

## Data Availability

Not applicable.
